# Knowledge, Awareness and Attitude on HPV, HPV Vaccine and Cervical Cancer among the College Students in India

**DOI:** 10.1371/journal.pone.0166713

**Published:** 2016-11-18

**Authors:** Shazia Rashid, Satyanarayana Labani, Bhudev C. Das

**Affiliations:** 1 Amity Institute of Biotechnology, Amity University Uttar Pradesh, Noida, India; 2 Department of Epidemiology and Biostatistics, Institute of Cytology and Preventive Oncology (ICPO), Uttar Pradesh, Noida, India; 3 Stem Cell & Cancer Research Lab, Amity Institute of Molecular Medicine & Stem Cell Research (AIMMSCR), Amity University Uttar Pradesh, Noida, India; Bharathidasan University, INDIA

## Abstract

**Background:**

Infection of specific high risk Human papillomaviruses (HPVs) is known to cause cervical cancer and two prophylactic vaccines have been developed against two major high risk HPV types 16 and 18 for prevention of cervical cancer. Because of societal, religious and ethical issues associated with the vaccination of adolescent girls in India together with lack of awareness about HPV and HPV vaccines, no successful HPV immunization program has been employed in India.

**Objective:**

To determine knowledge, awareness and attitude of college students on HPV, HPV vaccine and cervical cancer.

**Method:**

A questionnaire-based survey was conducted in a total of 1580 undergraduate students between the age group 16–26 years comprising 684 girls and 876 boys.

**Results:**

Out of a total of 1580 students, girls had more knowledge about cervical cancer (82.45%, p<0.001), HPV (45.61%, p<0.001) and HPV vaccines (44%, p<0.001) when compared to those in boys. However, knowledge about the types of HPV and vaccines was poor. Interestingly, students from biology-major had more knowledge and awareness about cervical cancer (81.89%, p<0.001) and HPV (46.58%, <0.001) when compared to non-biology students. Girls from both biology and non-biology group had higher awareness compared to boys. Analysis of odds ratio (ORs) along with 95% CI showed older girls with 1.2 to 3 fold (p<0.05) higher knowledge than boys. All students agreed that girls should get vaccinated against HPV (p<0.001).

**Conclusion:**

It is suggested that there is a need for educational intervention and awareness campaigns to augment HPV immunization program for control of cervical cancer in India.

## Introduction

Cervical cancer is the third most commonly diagnosed cancer worldwide and the fourth leading cause of cancer death in women [1] but in India, it is the leading cancer among women [2] accounting for more than 74,000 deaths per year [3].

The principal causative agent for the development of cervical cancer is the infection of specific high risk types of human papillomaviruses (HPVs). Though more than 140 types of HPV have been identified only about 40 types are sexually transmitted. Of these, two high risk HPV types 16 and HPV 18 are responsible for more than 80% of cervical cancer in India [4–7]. In addition, a variety of clinic-epidemiological risk factors such as early age of marriage, multiple sexual partners, multiple pregnancies, poor genital hygiene and smoking etc. are often associated with the development of cervical cancer [8]. For primary prevention, recently two vaccines, a quadrivalent (HPV 16, 18, 6 and 11) ‘Gardasil’ and a bivalent (HPV 19 and 18)‘Cervarix’ [9–10] have been introduced for vaccinating young adolescent girls between ages 9–13 and/or 13–26 year young adults[11]. These two HPV vaccines were US FDA (Food and Drug Administration) approved and are commercially available in India. However, inspite of these vaccines are reported to be highly immunogenic, safe, well tolerated and highly effective in preventing HPV infection, HPV vaccines are still not a part of the National Immunization Program. These vaccines were rather suspended because of certain unrelated deaths occurred during HPV vaccine immunogenicity trials conducted in the states of Andra Pradesh and Gujarat by PATH (Program for Appropriate Technology in Health) and ICMR (Indian Council of Medical Research) [12]. So no successful immunization program is introduced in India. There also exist other reasons such as higher cost of vaccines, including societal, religious and cultural issues and fear of adverse effects that may occur after immunization. Another major reason is the lack of awareness and knowledge about HPV, HPV vaccines and cervical cancer among people resulting in only small number of immunizations being carried out at private clinics. It is of general observation that HPV immunization of adolescent girls is mainly found among the educated mass of India. On the contrary, in western countries like U.S.A and Europe, HPV vaccines are approved for mass scale use and included in the national immunization program for reducing the burden of cervical cancer.

The success and benefit of control and prevention of cervical cancer largely depend to a great extent on the level of awareness and knowledge about different aspects of the disease and the vaccine. It is therefore important to target immunizable young adult college-going girls and boys, as both are part of the infection chain and at risk for HPV infection as they are living a more independent lifestyle but have a choice to undergo vaccination with the consent from parents and are within the age group of successful vaccination outcome. Hence, assessment of their knowledge, awareness and attitude towards the causes of cervical cancer, HPV infection and vaccination available for the disease including adequate participation in immunization programs can lead to successful reduction in disease burden and control of cervical cancer in India. Along with screening, HPV detection and prevention strategies, creating knowledge and awareness among youth can help in further reduction in cervical cancer burden. Thus, the study aimed at assessing the extent of knowledge, awareness and attitude towards HPV, HPV vaccine and cervical cancer among college students in a premier private university where students come from different parts of the country but mostly from a higher echelon of the society. The results demonstrate that the awareness and knowledge of HPV, HPV vaccine and cervical cancer is low and depend mainly on age, sex and education-background of the college students.

## Materials and Methods

### Study area and subjects

The study was carried out from January to March 2015 at Amity University Uttar Pradesh (AUUP), NOIDA campus which is one of the largest private universities of India. It is one of the biggest campuses and is located in the National Capital Region (NCR). It has students from all over India and abroad, the majority of whom belong to affluent and educated families with access to print and visual media. The students were from both urban and rural areas but majority belong to urban areas. The study was approved by the Institutional Ethics Committee, Amity University, NOIDA, India. An informed, written consent was obtained from each participant and recorded for the study. The informed consent form was also approved by the Institutional Ethics Committee, Amity University, NOIDA, India.

A pre-tested questionnaire (Table 1) was used in a cross-sectional study. The study was conducted in a total of 1580 undergraduate students comprising 684 girls and 876 boys between the age group of 16–26 years.

**Table 1:** Questionnaire employed for awareness about cervical cancer and HPV.

Q1	Do you know what uterine cervical cancer is?
Q2	What are the causes of cervical cancer?
Q3	What is Human papillomavirus (HPV) and its relation with cervical cancer?
Q4	What are the major cancer causing HPV types?
Q5	Does HPV cause genital warts in women?
Q6	Do you know there exists vaccines against HPV?
Q7	Do you know the names of HPV vaccines?
Q8	Who are eligible to take HPV vaccine?
Q9	Are you vaccinated for HPV
Q10	Should only adolescent girls take HPV vaccine?

### Sample size and questionnaire

Considering 25 percent HPV awareness with 3 percent allowable error and 95 percent CI, a sample of 800 was required. This was doubled i.e. 1600 to make it sufficient for sub-groups comparison. The pre-tested questionnaire was developed first to gather information on age, sex, religion, biology or non-biology background, place of residence and family income of the students while the second part formed a set of ten questions involving knowledge of cervical cancer, HPV and awareness about HPV vaccine and opinion about vaccination for girls and boys. The questionnaire was administered to the students and the data from questionnaire was processed anonymously by assigning random codes.

Students were categorised into groups based on three factors; sex (male vs. female), age (younger students ≤ 19years vs. older students > 19years) and educational background (biology vs. non-biology)in order to examine which of these factors are strongly associated with the knowledge, awareness and attitude towards cervical cancer, HPV and HPV vaccination. Biology background students were pursuing undergraduate education in Biotechnology and non-biology background students were from various streams of Engineering.

### Statistical analyses

Chi-square and Fishers’ exact tests were used to evaluate the association among various factors related to awareness cervical cancer and HPV. Statistical analysis was carried out using Statistical Package for Social Sciences program (SPSS, version 21.0, IBM). Multivariate analysis was applied and odds ratios (ORs) along with 95% CI were also calculated.

## Results

Data collected through questionnaire were tabulated and analysed using various statistical methods. Out of a total of 1580 students interviewed, the number of girls was 684 and 876 boys who were again categorised based on their age into younger (≤ 19years) and older (> 19years) and background in biology or physical sciences. Of the total students, girls had more knowledge about cervical cancer (82.45%, p<0.0001), causes of cervical cancer (11.54%, p<0.001) and HPV being the major cause of cervical cancer (45.61%, p<0.001) and also about genital warts (42.54%, p<0.001) compared to boys (Table 2). Among girls, older girls were more aware compared to younger girls about cervical cancer (88.55%, p<0.0001) and HPV as its major causative agent (57.23%, p<0.0001)(Table 2).There was no significant difference in awareness about oncogenic HPV types between girls and boys in general although a small percentage of older girls were more aware (2.02%, p<0.0001) than the younger girls about the oncogenic HPV types. Among boys, older boys had more knowledge about the cervical cancer (64.45, p<0.001), HPV as the major cause of cervical cancer (33.52%, p<0.001) and also responsible for genital warts (38.15, p<0.001)(Table 2). It was also observed that girls were more aware about the existence of HPV vaccines (44%, p<0.001) and eligibility of vaccination (6.28%, p<0.001) compared to boys. However, knowledge about the names of the vaccines was low in both girls and boys. Among girls, older girls were more aware about the existence of HPV vaccines (52.52%, p<0.001). In response to a question regarding mandatory HPV vaccination for girls, significant number of girls responded positively compared to boys (73.24%, p<0.001). A good percentage of older boys also responded positively and were in favour of girls getting vaccinated against HPV (63.58%, p<0.05)(Table 2).

**Table 2:** Knowledge about cervical cancer and HPV among college students based on their sex.

Parameters responded positive		Girls	Young girls		Older girls		Boys	Younger boys		Older boys	
		n = 684	n = 387		n = 297		n = 876	n = 549		n = 346	
		No. (%)	No.	%	No.	%	No. (%)	No.	%	No.	%
Q1	Do you know what uterine cervical cancer is?	564 (82.45)***	301	**77.77**	263	88.55***	**500 (57.07)**	290	**52.82**	223	64.45***
Q2	What are the causes of cervical cancer?	79 (11.54)***	33	**8.52**	46	15.48***	**59 (6.73)**	28	**5.10**	32	9.24*
Q3	What is Human papillomavirus (HPV) and its relation with cervical cancer?	312 (45.61)***	142	**36.69**	170	57.23***	**242 (27.62)**	135	**24.59**	116	33.52***
Q4	What are the major cancer causing HPV types?	**7 (1.02)**	1	**0.25**	6	2.02**	**13 (1.48)**	7	**1.28**	6	**1.73**
Q5	Does HPV cause genital warts in women?	291 (42.54)***	148	**38.24**	143	48.14***	**272 (31.05)**	147	**26.78**	132	38.15***
Q6	Do you know there exists vaccines against HPV?	301 (44.00)***	145	**37.46**	156	52.52***	**273 (31.16)**	158	**28.78**	121	**34.97**
Q7	Do you know the names of HPV vaccines?	**13 (1.90)**	4	**1.03**	9	**3.03**	**12 (1.36)**	9	**1.64**	3	**0.87**
Q8	Who are eligible to take HPV vaccine?	43 (6.28)***	15	**3.87**	28	9.42**	**16 (1.82)**	6	**1.09**	10	2.89*
Q9	Are you vaccinated for HPV	49 (7.16)***	28	**7.24**	21	**7.07**	**65 (7.42)**	45	**8.20**	21	**6.07**
Q10	Should only adolescent girls take HPV vaccine?	501 (73.24)***	270	**69.76**	231	77.77*	**519 (59.24)**	309	**56.28**	220	63.58*

Younger (≤19 years) and older (>19 years);

* p<0.05,

** p<0.01,

***p <0.001

The students were also categorised into two groups based on their educational subject specialization such as biology and non-biology groups (Table 3). Biology students showed significantly higher knowledge about cervical cancer (81.89%, p<0.001) and HPV as its major causative agent (46.58%, p<0.001) compared to non-biology students. Both among, biology and non-biology students, girls were more aware compared to boys (87.06% &65.03%; p<0.001, respectively). About 43.62% (p<0.001) of students with biology background were aware about the HPV vaccines but did not know about the names of the vaccines. The non-biology background students in general had low knowledge and awareness about HPV, its role in causing cervical cancer and HPV vaccine. However, the overall attitude towards girls getting vaccinated against HPV was high in both biology and non-biology students (72.20%, p<0.001; 68.53%, p<0.001).

**Table 3:** Knowledge about cervical cancer and HPV among college students based on their academic subject background.

Parameters responded positive		Biology background	Girls		Boys		Non-biology background	Girls		Boys	
		n = 878	n = 541		n = 337		n = 702	n = 143		n = 558	
		No. (%)	No.	%	No.	%	No. (%)	No.	%	No.	%
Q1	Do you know what uterine cervical cancer is?	719 (81.89)***	471	87.06***	248	**73.59**	**358 (50.99)**	93	65.03***	265	**47.49**
Q2	What are the causes of cervical cancer?	110 (12.52)***	73	**13.49**	37	**10.98**	**29 (4.13)**	6	**4.20**	23	**4.12**
Q3	What is Human papillomavirus (HPV) and its relation with cervical cancer?	409 (46.58)***	277	51.20***	132	**39.17**	**154 (21.93)**	35	**24.48**	119	**21.33**
Q4	What are the major cancer causing HPV types?	**9 (1.02)**	7	**1.29**	2	**0.59**	**11 (1.56)**	0	**0.00**	11	**1.97**
Q5	Does HPV cause genital warts in women?	376 (42.82)***	248	45.84*	128	**37.98**	**194 (27.63)**	43	**30.07**	151	**27.06**
Q6	Do you know there exists vaccines against HPV?	383 (43.62)***	259	47.87**	124	**36.80**	**197 (28.06)**	42	**29.37**	155	**27.78**
Q7	Do you know the names of HPV vaccines?	**17 (1.93)**	13	**2.40**	4	**1.19**	**8 (1.13)**	0	**0.00**	8	**1.43**
Q8	Who are eligible to take HPV vaccine?	52 (5.92)***	41	7.57**	11	**3.26**	**7 (0.99)**	2	**1.40**	5	**0.90**
Q9	Are you vaccinated for HPV	43 (4.89)***	37	6.83***	6	**1.78**	**72 (10.25)**	12	**8.39**	60	**10.75**
Q10	Should only adolescent girls take HPV vaccine?	634 (72.20)***	403	**74.49**	231	**68.55**	**396 (56.41)**	98	68.53***	298	**53.41**

* p<0.05,

** p<0.01,

***p <0.001

Overall, older students with background in biology showed more knowledge and awareness about cervical cancer as being the most common cancer in women and HPV as its primary causative agent compared to the younger non-biology or biology students. Multiple logistic regression analysis with respect to age, sex and educational-background resulted in adjusted odds ratio with 95% CI that showed older girls with biology-background had 1.2 to 3-fold higher knowledge about cervical cancer (p<0.05) compared to younger boys with non-biology-background (Table 4). Older students with biology-background had significant knowledge about the general causes of cervical cancer and HPV being one of the major causes of this cancer (p<0.05). Older students had significantly higher knowledge (OR:2.9, p<0.05) about the type of oncogenic HPV when adjusted with sex, age and the subject group to which they belong. Older girls with biology had 1.3 to 1.4-fold higher knowledge about availability of HPV vaccines and 1.9 to 3.4-fold higher knowledge about eligibility for HPV vaccine (p<0.05). However, there was no difference in the knowledge about the names of HPV vaccines available commercially between boys and girls. Girl students with biology background were in favour of getting HPV vaccination for young adolescent girls (OR:1.5, p<0.05).

**Table 4:** Adjusted odds ratio (ORs) along with 95% CI for various aspects of knowledge on cervical cancer and HPV among college students.

Parameters	Knowledge about cervical cancer	Cause of cervical cancer	Knowledge about HPV and relation with cervical cancer	Knowledge about oncogenic HPV types	Knowledge about HPV causes genital warts	HPV vaccine availability awareness	Knowledge about the names of HPV vaccines	Knowledge about eligibility for vaccine	HPV vaccination willingness for girls
**Older age—OR (*p value*)**	1.235** (0.011)	1.479* (0.039)	1.458*** (0.001)	2.913* (0.030)	1.393** (0.004)	1.356** (0.008)	1.176 (0.705)	1.960* (0.020)	1.224 (0.088)
**(95% CI)**	(0.95, 1.59)	(1.01, 2.16)	(1.15, 1.83)	(1.08, 7.80)	(1.10, 1.74)	(1.07, 1.70)	(0.50, 2.79)	(1.09, 3.50)	(0.96, 1.54)
**Female Sex—OR (*p value*)**	2.303*** (0.000)	1.268 (0.223)	1.556*** (0.000)	0.927 (0.885)`	1.357*** (0.000)	1.446** (0.002)	1.209 (0.668)	2.443** (0.005)	1.567*** (0.000)
**(95% CI)**	(1.76, 3.00)	(0.85, 1.87)	(1.22, 1.97)	(0.32, 2.65)	(1.07, 1.71)	(1.14, 1.82)	(0.49, 2.92)	(1.29, 4.59)	(1.22, 1.99)
**Biology—OR (*p value*)**	3.004*** (0.000)	2.634*** (0.000)	2.304*** (0.000)	0.462 (0.151)	1.552*** (0.000)	1.544*** (0.001)	1.497 (0.419)	3.392** (0.006)	1.573*** (0.000)
**(95% CI)**	(2.30, 3.91)	(1.62, 4.27)	(1.77, 2.98)	(0.15, 1.35)	(1.2, 1.99)	(1.19, 1.98)	(0.55, 4.06)	(1.39, 8.23)	(1.22, 2.01)

Older age (>19 years),

* *p*<0.05,

** *p*<0.01,

***p<0.001;

CI: Confidence Interval

## Discussion

Cancer of the uterine cervix is a major cancer in India in women but it is a curable cancer, if detected early. The two major high risk oncogenic HPV genotypes, HPV16 and HPV 18 most commonly associated with the development of cervical cancer, are highly prevalent in India [4–7]. Integrating knowledge and awareness programs with educational intervention for cervical cancer or HPV screening along with HPV vaccination will go a long way in reducing HPV infection and controlling cervical cancer in Indian women. The present study was carried out in undergraduate science and engineering students in an affluent private university set up with the main aim of getting information regarding the level of awareness and knowledge about cervical cancer, HPV and attitude towards HPV vaccination that may help in designing effective HPV screening and successful vaccination programs for the control of cervical cancer. We observed a close association between increasing age and higher HPV awareness especially among the girls. In general, girls who have passed through their teenage and from biology-background were more knowledgeable than others about cervical cancer, its causes and HPV being the major causative agent for cervical cancer (Tables 2 & 3). However, none of the students were aware about the cancer causing HPV types and names of the HPV vaccines which reflect that they have a very limited knowledge and understanding of the disease. It was also observed that although students of this University belonged to well-educated and affluent class families, the percentage of girls who got vaccinated was very low (7%). This is alarming because even after almost a decade of introduction of HPV vaccines, which are available in India since 2008, not many parents are interested to get their daughters vaccinated. It appears that several factors involving societal, religious and prejudice ideas, socioeconomic status including lack of knowledge, awareness and attitude might be the reasons for such a low coverage of HPV vaccination in India. Several studies have been conducted to analyse the awareness about cervical cancer and HPV among young women, both in rural and urban areas of India [13–17] and in other countries [18–20]. All these studies report that awareness and knowledge about cervical cancer is very poor among both young undergraduate and postgraduate students, from rural and urban areas which may be due to lack of education and exposure to print/ audio-visual media, including variety ofsocietal factors, social stigma and ignorance [21–23]. However, a study carried out earlier in undergraduate medical students in India show that they are well aware about the cervical cancer and its link to HPV infection [24]. In general, girls have shown more awareness compared to boys (Table 2) and the same has also been reported recently by Hussain et al.[21]. The level of awareness among students showed a strong association with the educational background in biology than in non-biology. Biology students were more aware about cervical cancer, HPV and HPV vaccination compared to non-biology students (Table 3). The study highlights the need to improve public health education in view of the lack of awareness about cervical cancer and its possible prevention by vaccination in the community [25]. Studies have shown that imparting education has positive effect in encouraging and motivating men and women to participate in screening and vaccination programs. It has been reported recently that a brief educational intervention could increase college students’ performance on an HPV knowledge assessment from 45% to 79% 3 months later [26]. Not only can this lead to early diagnosis of the disease but also successful implementation of HPV immunization programs for control of cervical cancer.

The level of awareness among boys was found to be poor as compared to girls who were more aware about cervical cancer and HPV (Table 2).A study conducted among Australian men and women showed similar results with higher awareness among young women compared to men [27]. In a study carried out on 18- to 25-year-oldsin six vocational schools in Berlin, it was found that 50% women were aware about HPV compared to only 25% of the men [28]. Similar results have been found among developing countries as well [29]. Analysis of multiple factors also showed that older girls with biology background were more aware/ knowledge about HPV and HPV vaccination (Table 4).

In the present study, both boys and girls showed positive attitude towards girls getting vaccinated against HPV after knowing that it can provide protection against HPV that causes cervical cancer (Table 2). HPV immunization program in western countries is highly successful due to widespread vaccination drives and awareness programs initiated by the government and healthcare organisations. In U.S.A. in 2014, 39.7% adolescent girls aged 13–17 yr received all three doses of HPV vaccine [30]. HPV vaccination and compliance rate of 80% have been reported from Great Britain and Portugal in 2012 [31]. But in India where a majority of population is positive for high risk oncogenic HPV types; specifically HPV type 16 and prevalence of cervical cancer and associated mortality is one of the highest in the world [32–37], no national HPV immunization programs have been initiated till date. Although, a small HPV vaccine immunogenicity trials were initiated by PATH in collaboration with ICMR, in the states of Andra Pradesh and Gujarat in India but these were stopped in the initial stage due to some unrelated deaths, medical, safety and ethical issues. HPV vaccination in India is therefore restricted to only private clinics and hospitals and it has been observed that vaccine acceptability is mainly restricted among the educated mass. Majority of the studies have shown that most of participants or parents of participants are unwilling to get their daughters vaccinated due to lack of knowledge of HPV vaccine, its safety and efficacy, including the socio-cultural, economic and religious issues. Many are of the opinion that HPV vaccines would make sex safe, leading to promiscuity and change in sexual behaviour in younger generation, which would cause social stigma, and may tarnish family prestige [21, 38–39]. Hence, there is an imperative need for educational intervention to raise knowledge and awareness for HPV associated cancers and their prevention by vaccination in young and adolescent girls, de-stigmatize HPV infection and for successful implementation of screening and vaccination programs in order to reduce mortality and the burden of cervical cancer. It may also control high risk HPV associated other diseases such as head and neck cancer, oesophageal cancer etc. that are highly prevalent in India.

## Conclusion

The study suggests that the overall awareness and knowledge about cervical cancer, HPV and HPV vaccination are poor among college students and are strongly associated with the age and educational background of the students in biology. There is an urgent need for educational intervention—both formal and informal, not only for girls and boys from both biology and non-biology background but also for parents to change their attitude towards HPV vaccination. To achieve this, both print and audio-visual media may play a pivotal role. It is suggested that there is a need for incorporation of HPV vaccination in the National Immunization Program which can be integrated with the screening (VIA/ Pap/ HPV DNA) and national cancer control program, and eliminate the limitations of vaccine cost and poor awareness for successful control of HPV- induced cervical and other cancers.

## Supporting Information

S1 File Original raw data. (XLSX)

## Abbreviations

HPV: Human papillomaviruses
CI: Confidence interval
VIA: Visual inspection of cervix with acetic acid
